# Supplementary material for: Genetic interactions derived from high-throughput phenotyping of 6589 yeast cell cycle mutants
Source: NPJ Syst Biol Appl. 2020 May 6;6:11. doi: 10.1038/s41540-020-0134-z (PMC7203125; doi:10.1038/s41540-020-0134-z)
Supplement: Supplementary file 7 — Supplementary Information [file 41540_2020_134_MOESM7_ESM.pdf]

Genetic interactions derived from high-throughput phenotyping of 6,589 yeast cell cycle mutants

Supplemental Methods and Figures

Jenna E Gallegos, Neil R. Adames, Mark Rogers, Pavel Kraikivski, Aubrey Ibele, Kevin Nurzynski-Loth, Eric Kudlow, T.M. Murali, John J. Tyson, Jean Peccoud

Supplementary Methods

Parent Strain Construction

We used several strategies to generate the eight sets of parent strains used in this study (Table S1). We obtained most of the Set 1 and Set 3 strains by sporulation and tetrad analysis of the heterozygous diploid commercial collection of *kanMX* strains [1,2], but we made some by *de novo kanMX* PCR-mediated gene deletions in BY4741 or BY4742 (using pFA6a-kanMX as the template and listed primers[3-6], Table S2). We obtained most of the Set 2 and Set 4 strains by transformation of the heterozygous diploid strains with a *natMX* PCR product (using MX.for and MX.rev primers in Table S2 with pAG25 template. pAG25 and its sequences are available from Addgene) to switch markers, followed by selection of nourseothricin-resistant/G418-sensitive transformants, sporulation, and tetrad analysis. We made the rest of sets 2 and 4 by *de novo natMX* PCR-mediated gene deletions in BY4741 or BY4742 (using pAG25 as the template and listed primers). We made most of Set 7 and Set 8 by *de novo* PCR-mediated gene deletions in the SGA strain Y8205 [7], but we made some of these strains by crossing one of the BY4741-derived gene deletion strain with Y8205, followed by tetrad dissection. We then used the SGA method to cross these strains to BY4741 and obtain *MAT***a** versions of these strains for Set 5 and Set 6. All strains were confirmed by PCR of genomic DNA using one set of test primers for the gene deletion and another for the wild-type gene[8] (Table S2). All strains (parents and progeny) are available upon request.

Double Mutant Progeny Construction

All crosses followed a standard format in which the *MAT*α strains (Sets 3, 4, 7, and 8) which we will call the "hit" strains, were arrayed alphabetically by gene name so that each strain was a single well in a 36-well block, with two replicate *MAT*α blocks per plate, leaving the first and fifth rows empty for the addition of the wild-type parents during phenotyping. If a deletion strain was missing in a *MAT*α set, we left the position empty. We arrayed the *MAT***a** strains, which we will call the query or "bait" strains, so that each *MAT***a** strain in the set fills a block of 36 wells at the same positions as one of the two blocks of *MAT*α strains (i.e., in rows 2-4 or 6-8).

Before crossing, each set of parent strains was arrayed in 96-well microtiter PlusPlates (Singer Instruments, Somerset, UK) containing YPD broth and pinned onto YPD+G418 (300 µg/ml; odd numbered sets) or YPD+nat (150 µg/ml; even numbered sets) and grown for 3-4 days at 30 °C.

For the crosses, we used a Rotor HDA (Singer Instruments, Somerset, UK) to replica-pin each *MAT*α plate to 12-18 YPD plates using 96 long repads with 6 wet mix cycles and 4 dry mix cycles to ensure robust inoculation of each plate. Visual inspection of each plate ensured proper transfer of cells. We then pinned each *MAT***a** plate on top of one *MAT*α plate using the same conditions to ensure good mixing of the two parent strains on the YPD plate. Matings were performed on YPD at 30 °C for two days.

Diploids were selected on YPD + G418/nat (300/150 µg/ml) at 30 °C for two days. Diploids were sporulated on enriched sporulation media (1% potassium acetate, 0.1% yeast extract, 0.05% glucose, 0.1 g of his/leu/lys/ura supplement) at 24 °C for five days.

Haploid progeny were selected as described in Tong et al. (2006) [7], except that we separately selected for both *MAT***a** and *MAT*α progeny. We first selected haploids from the sporulation plates by replica-pinning on SD-arg/his/lys+canavanine/thialysine (100/100 µg/ml) for *MAT***a** progeny and SD-arg/leu/lys+canavanine/thialysine (100 µg/ml) for *MAT*α progeny. For the second round of haploid selection, we added G418 (300 µg/ml) to these plates. For the final haploid selection, we added both G418 (600 µg/ml) and nourseothricin (150 µg/ml) to obtain double mutant haploids.

Halo Assays

Halo assays were used to confirm the mating type of the parents and progeny and identify potential cell signaling and chromosome segregation defects**.** We performed halo assays as described [9] using Y955 lawns to test for **a**-halos, and Y991 to test for α-halos. We prepared halo assay plates by growing Y995 and Y991 in YPD broth at 30 °C overnight in a shaking incubator. The next morning, we diluted each strain 1/5 in YPD broth, vortexed the tubes, and used sterile glass beads to spread 500 µl of the dilution per plate onto YPD PlusPlates. We allowed these plates to dry before pinning each plate of parent strains or haploid progeny (at 96 colony densities) onto both a Y995 lawn (to test for **a**-factor secretion) and a Y991 lawn (to test for α-factor secretion). We imaged halo assay plates after 48 h of growth (see Additional Data). Individual lines that did not behave as expected (most likely due to isolated genetic mishaps) were not excluded, as the consensus between multiple biological replicates was used to draw the conclusions presented.

Identifying Curation Errors

For several of the manually curated synthetic lethal interactions on the Saccharomyces Genome Database, we found that the listed SL interaction was not supported by the cited paper. In some cases, what was curated as synthetic lethal was in fact not lethal but exhibited some other kind of growth defect. In other cases, the interaction was lethal, but was tested in a mutant background in which one or more additional cell cycle genes were knocked out. Those synthetic lethal interactions that we found to be curation errors are listed, along with their references, below:

clb5Δ clb6Δ[10-12], *cdc55Δ cln1Δ*[13], *cdc55Δ cln2Δ*[13], *cln1Δ cln2Δ*[14-20], *cln1Δ cln3Δ*[16,17], *cln1Δ msn5Δ*[21], *cln2Δ cln3Δ*[16,17], *cln2Δ msn5Δ*[21], *fkh1Δ fkh2Δ*[22], *ssa1Δ ydj1Δ*[23]

Tetrad Analysis

To identify synthetic lethality by tetrad analysis, several tetrads (usually 12) were dissected for one or more biological replicates of each of the 58 gene combinations listed in Figure 4. The surviving spores were patched onto YPD plates, and then replica plated onto YPD+G418 (600ug/ml), YPD+nat (150ug/ml), and Y995 and Y991 lawn plates (as described above but using 300ul diluted culture).

Dissections from which we could recover very few live spores of any genotype were identified as having a likely meiotic defect (MD). Recovery of a live spore that was resistant to both antibiotics was considered evidence for viability (V). Cases where a dead spore in a tetrad could be inferred to have the double mutant phenotype based on allele segregation were considered evidence for synthetic lethality (SL). If the ratio of spores supporting synthetic lethality to spores supporting viability (SL:V) was 4:1 or greater, we considered the gene combination SL. If the SL:V ratio was between 4:1 and 1:1, we considered the gene combination to have reduced viability (RV). If the SL:V ratio was less than 1:1, we considered the gene combination to be viable (V). Cases where we were able to identify fewer than two spores as potentially SL or V (due to low viability overall) were also designated MD.

Supplementary Figures

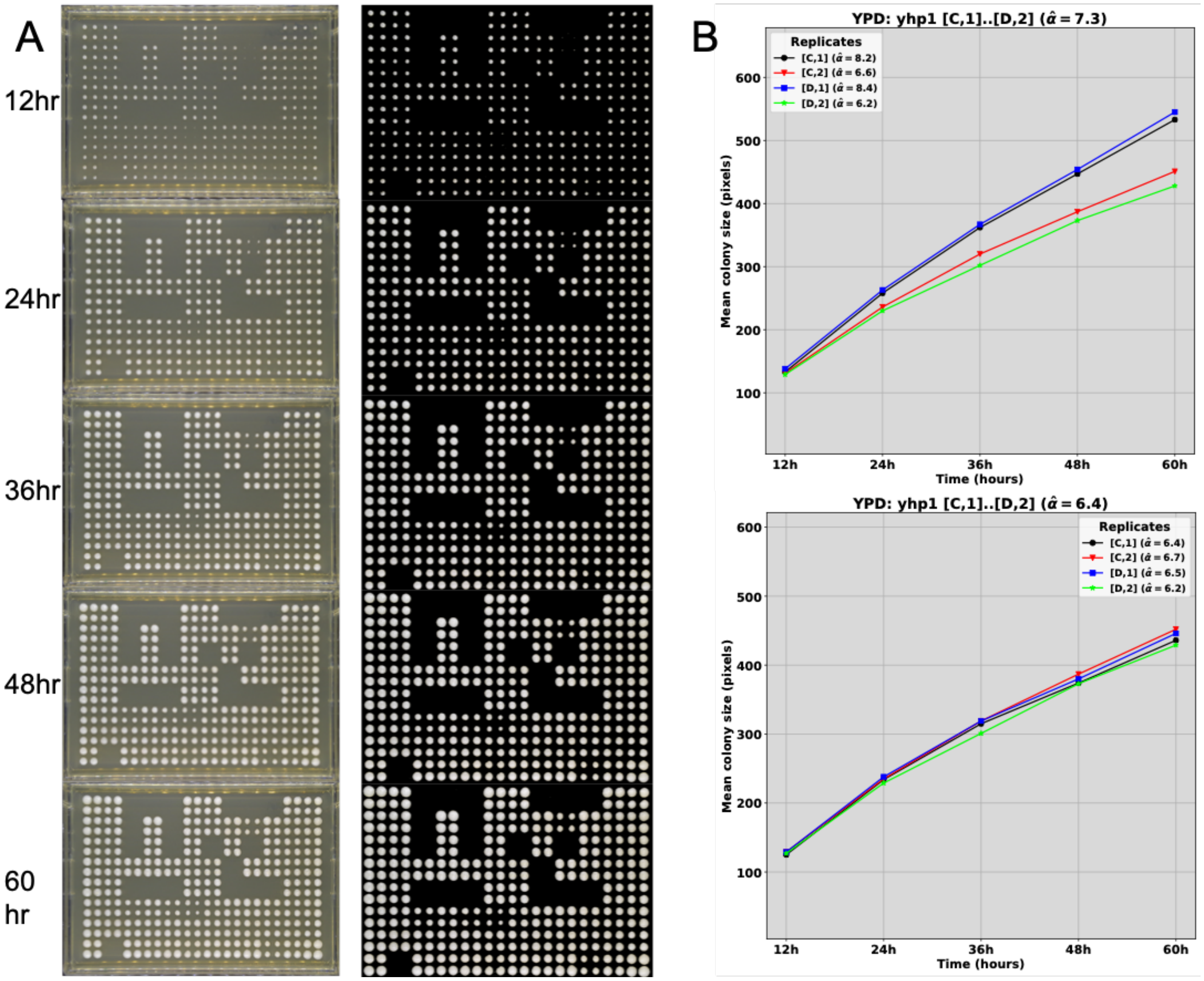

Figure S1. Example of plate images and growth curves. (A) Unprocessed (left) and processed (right) images of a phenotyping plate across the 5 time points. **(B)** Growth curves for one of the quadruplicates shown in A before (top) and after (bottom) normalization. Each colored line represents one of the colonies in the quadruplicate. The black and blue lines plot edge colonies (positions C1 and D1), while the red and green lines plot non-edge colonies (C2 and D2).

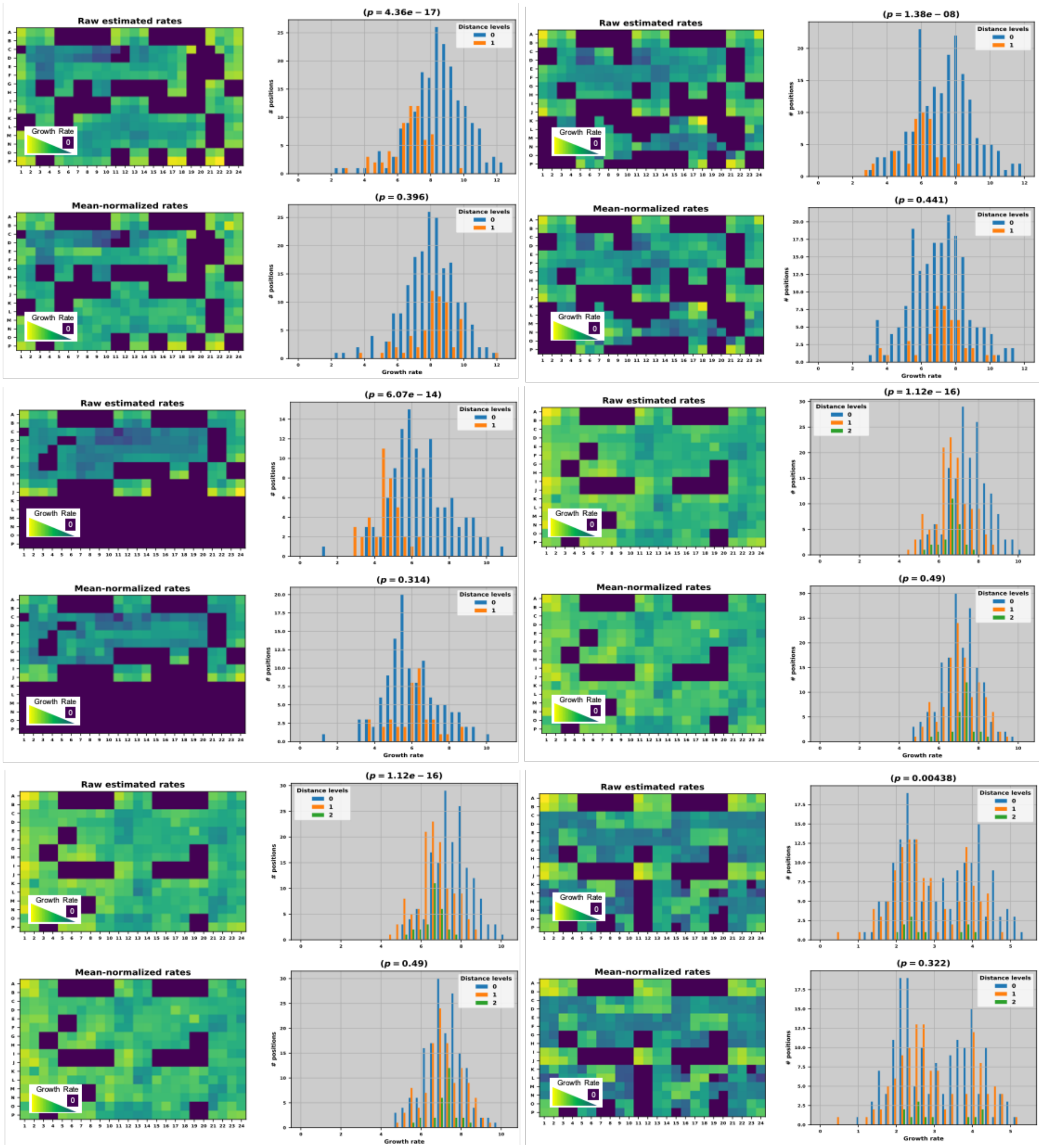

Figure S2. Normalizations for six representative phenotyping plates. Normalizations based on the mean growth rate of the wildtype controls on each plate were used to account for edge effects. Heat maps show a visual representation of growth rates across each plate. The X and Y axis are the coordinates for the 384 positions where a colony may appear. In every case, wild-type controls are in rows A, B, I, and J, columns 1-4, 11-14, and 21-24. Histograms compare the growth rate of colonies that are on the edge of the plate or adjacent to an empty position (distance level 0) with those that are one or more positions away from an edge (non-zero distance levels). The p-value reported above the histogram marks the significance of the difference between the growth rate of edge-adjacent colonies and internal colonies. In each case, raw, unnormalized heat maps, histograms, and p-values are shown just above their normalized counterparts.

Figure S3: Basal parameter values for wild-type cells in the 2020 model. Parameter values differing from the 2015 model are highlighted.

**Rate constants** (min^{-1}) (subscripts: “s” for synthesis, “d” for degradation)							
$k_{s,bck2}$	0.13	$k_{d,bck2}$	0.25				
$k_{s,bud,e}$	1.3	$k_{d,bud}$	0.01				

$k_{s,cdc20}$	0.0008	$k_{s,cdc20,m1}$	0.2	$k_{d,cdc20}$	0.32		
$k_{s,cki}$	0.00124	$k_{s,cki,swi5}$	0.02745	$k_{d,cki}$	0.0153	$k_{d,ckip}$	0.5
$k_{s,clb2}$	0.0052	$k_{s,clb2,m1}$	0.19772				
$k_{d,clb2}$	0.0086	$k_{d,clb2,20}$	1.1642	$k_{d,clb2,20,i}$	0.15	$k_{d,clb2,h1}$	0.6
$k_{s,clb5}$	0.0008	$k_{s,clb5,mbf}$	0.0156				
$k_{d,clb5}$	0.035	$k_{d,clb5,20}$	0.8	$k_{d,clb5,20,i}$	0.25		
$k_{s,cln2}$	0	$k_{s,cln2,sbf}$	0.1	$k_{d,cln2}$	0.135		
$k_{s,cln3}$	0.1	$k_{d,cln3}$	0.2				
$k_{s,ori,e}$	2	$k_{d,ori}$	0.06				
$k_{s,pds1}$	0.03	$k_{s,pds1,mbf}$	0.03				
$k_{d,pds1}$	0.01	$k_{d,pds1,20}$	2.5	$k_{d,pds1,20,i}$	1.7		
$k_{s,polo}$	0	$k_{s,polo,m1}$	0.22	$k_{d,polo}$	0.3	$k_{d,polo,h1}$	1.5
$k_{s,swi5}$	0.005	$k_{s,swi5,m1}$	0.03	$k_{d,swi5}$	0.08		
$k_{s,spn}$	0.24	$k_{d,spn}$	0.03				
$k_{s,swe1}$	0.001	$k_{s,swe1,sbf}$	0.007				
$k_{d,swe1}$	0.01	$k_{d,swe1,h1}$	0.5	$k_{d,swe1p}$	0.2	$k_{d,swe1p,h1}$	0.7
**Other time-scale factors** (min^{-1})							
μ	0.0077	(*mdt* = 90 min in glucose medium)					
γ	1	γ_{cki}	10	γ_{apc}	0.5	γ_{tem1}	0.1
**Interaction coefficients** (dimensionless)<br>(subscripts: "a" for activation, "i" for inactivation, "p" for phosphorylation, "dp" for dephosphorylation)							
$\omega_{a,apc,b2}$	0.625	$\omega_{i,apc}$	0.7				
$\omega_{a,bub2,14}$	0.05	$\omega_{a,bub2}$	2.71	$\omega_{a,bub2,c55}$	0.8	$\omega_{i,bub2,lo}$	4.4
$\omega_{i,bub2,lte,lo}$	6.7						
$\omega_{a,cdc15,14}$	0.85	$\omega_{i,cdc15}$	0.23	$\omega_{i,cdc15,b2}$	0.0149		
$\omega_{i,cdc55,p1}$	0.981						
$\omega_{a,cdh1,14}$	1.1	$\omega_{a,cdh1}$	0.032				
$\omega_{i,cdh1,n2}$	0.202	$\omega_{i,cdh1,b5}$	8.76	$\omega_{i,cdh1,b2}$	0.162		
$\omega_{p,cki,n2}$	1.15	$\omega_{p,cki,b5}$	9.5	$\omega_{p,cki,b2}$	1.65		
$\omega_{dp,cki}$	0.7	$\omega_{dp,cki,14}$	1.747				
$\omega_{dp,clb2}$	1.5	$\omega_{p,clb2,we}$	1.05				
$\omega_{a,mad2}$	30	$\omega_{i,mad2}$	0.6				
$\omega_{a,mcm1,b2}$	10	$\omega_{i,mcm1}$	1.7				
$\omega_{p,net1,b2}$	0.0225	$\omega_{p,net1,en}$	6.6	$\omega_{p,net1,15}$	0.288	$\omega_{p,net1}$	0.22
$\omega_{dp,net1}$	0.055	$\omega_{dp,net1,14}$	2.51	$\omega_{dp,net1,c55}$	1.0		
$\omega_{a,polo,b2}$	4.8	$\omega_{i,polo}$	0.2				
$\omega_{p,s6s4pw,n3}$	8.43	$\omega_{p,s6s4pw,n2}$	0.01	$\omega_{p,s6s4pw,k2}$	2.1	$\omega_{p,s6s4pw,b5}$	0.01

$\omega_{i,s6s4pw}$	0.8	$\omega_{p,s6s4pw,b2}$	2.87				
$\omega_{p,s6s4,n3}$	3.6	$\omega_{p,s6s4,n2}$	0.28	$\omega_{p,s6s4,k2}$	0.6	$\omega_{p,s6s4,b5}$	4.62
$\omega_{i,s6s4}$	0.5	$\omega_{p,s6s4,b2}$	0.035	$\omega_{p,s6s4np,b2}$	0.4		
$\omega_{p,s4s4p,k2}$	8.5	$\omega_{i,s4s4p}$	0.5	$\omega_{p,s4s4p,b2}$	4.5		
$\omega_{p,s6mb,n3}$	3.1	$\omega_{p,s6mp,n2}$	0.2	$\omega_{p,s6mb,k2}$	1.25	$\omega_{p,s6mb,b5}$	6.2
$\omega_{i,s6mb}$	0.5	$\omega_{dp,s6mb,b2}$	0.73				
$\omega_{p,ssa1,b2}$	2.0	$\omega_{dp,ssa1}$	1.0				
$\omega_{p,swe1,b2}$	1.5	$\omega_{dp,swe1}$	0.5				
$\omega_{a,swi5,14}$	5.1	$\omega_{a,swi5}$	0.2	$\omega_{i,swi5,b2}$	1.0		
$\omega_{a,tem1,lo}$	1.1	$\omega_{a,tem1}$	0.5	$\omega_{i,tem1,bub2}$	2.5		
**Total concentrations** (dimensionless)							
$[APC_T]$	25	$[Bub2_T]$	1	$[Cdc14_T]$	2	$[Cdc15_T]$	1
$[Cdc55_T]$	1	$[Cdh1_T]$	1	$[Esp1_T]$	0.5	$[Mad2_T]$	25
$[Mbp1_T]$	5.5	$[Mcm1_T]$	1	$[Net1_T]$	3.55	$[Pr_2]$	2
$[Pr_5]$	2	$[Ssa1_T]$	1	$[Swi4_T]$	5.5	$[Swi6_T]$	30
$[Tem1_T]$	2	$[Whi5_T]$	10				
**Other parameters** (dimensionless)							
$e_{bud,b5}$	0.38	$e_{bud,n2}$	0.45	$e_{bud,n3}$	0.3	$e_{ori,b2}$	0.35
$e_{ori,b5}$	0.5	J_{spn}	0.14	$k_{5,s6m}$	0.16	k_{s4}	0.91
k_{s6m}	0.63	k_{s4s6}	0.3	$k_{5,s6s4}$	1.1	k_{ydj1}	2.3
$r_{cdc14,n \to c}$	13	$r_{cdc14,c \to n}$	0.022	v_{bck2}	3	v_{clb2}	1.4
v_{clb5}	0.2	v_{cln3}	12	v_{ydj1}	2.9	θ_{cd}	0.2
θ_{cleave}	0.05	θ_{rl}	0.199	σ	10	$\rho_{14,net1}$	1

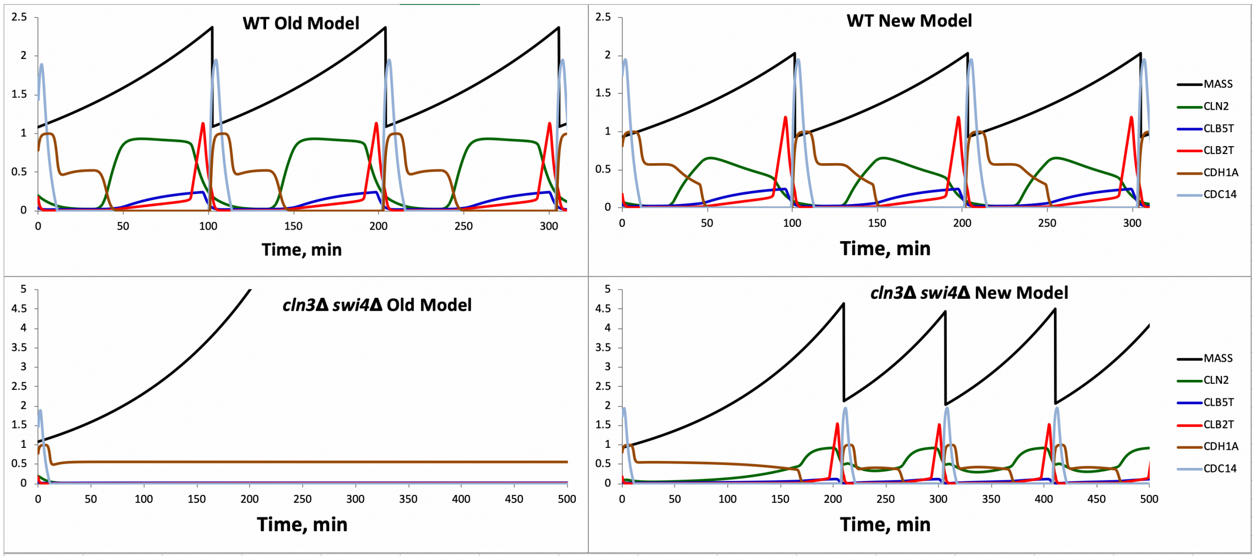

Figure S4: Comparison of the 2015 and 2020 model. Adjusting 13 parameters of the 2015 model does not significantly affect the dynamics of the model in the WT cells as shown by representing the time-evolution of variables (top half of the figure). However, the adjusted parameter values result in qualitative changes of the dynamics in selected mutants as shown in the bottom half of the figure.

Figure S5: List of mutant simulations. We have simulated all the mutants in this figure. We compared simulations and published phenotypes when possible. Mutants highlighted in red indicate inconsistencies between simulation and phenotypes. Mutants we have simulated for which no observed phenotype has been published are indicated with *. Mutants highlighted in bold have different phenotypes in the 2015 and 2020 models. New mutant simulations reported in the "New Data" column on the left.

New Data
bck2Δ cdh1Δ
bck2Δ cln3Δ
bck2Δ swi4Δ
bck2Δ swi6Δ
bfa1Δ lte1Δ
bub2Δ cdc55Δ
cdc55Δ cdh1Δ
cdc55Δ clb5Δ
cdc55Δ lte1Δ
cdc55Δ swi4Δ
cdh1Δ clb5Δ
cdh1Δ cln3Δ
cdh1Δ lte1Δ
cdh1Δ mad2Δ
cdh1Δ sic1Δ
cdh1Δ ssa1Δ
cdh1Δ swi4Δ
cdh1Δ swi5Δ
cdh1Δ swi6Δ
cdh1Δ whi5Δ
cdh1Δ ydj1Δ
clb1Δ clb2Δ
clb2Δ CLB1 mad2Δ
clb2Δ CLB1 swi6Δ
clb2Δ CLB1 sic1Δ
clb5Δ sic1Δ
clb5Δ swi4Δ
clb5Δ swi6Δ
cln3Δ swi4Δ
lte1Δ mad2Δ
lte1Δ sic1Δ
lte1Δ swi6Δ
lte1Δ ydj1Δ
mad2Δ sic1Δ
mad2Δ whi5Δ
mbp1Δ swi4Δ
swi4Δ ydj1Δ
swi4Δ swi6Δ

Wild-type
In glucose
In galactose

Start –mutants
mbp1Δ
ssa1Δ
swi4Δ
swi6Δ
swi6Δ in galactose
whi5Δ
ydj1Δ
WHI5^{OP}
bck2Δ mbp1Δ
bck2Δ whi5Δ
cln3Δ mbp1Δ
cln3Δ ssa1Δ
cln3Δ swi6Δ
cln3Δ whi5Δ
cln3Δ ydj1Δ
mbp1Δswi6Δ
mbp1Δ whi5Δ
swi4Δ whi5Δ
swi6Δ whi5Δ
whi5Δ GAL-BCK2
bck2Δ cln3Δ swi6Δ
bck2Δ cln3Δ whi5Δ
bck2Δ swi6Δ sic1Δ
*bck2Δ swi6Δ GAL-CLB5**
bck2Δ swi6Δ whi5Δ
*cln1Δ cln2Δ mbp1Δ**
cln1Δ cln2Δ swi4Δ
cln1Δ cln2Δ swi6Δ
*cln3Δ mbp1Δ swi6Δ**
cln3Δ mbp1Δ multi-copy *BCK2**
*cln3Δ mbp1Δ whi5Δ**
cln3Δ swi4Δ sic1Δ*
cln3Δ swi4Δ multi-copy *BCK2**
*cln3Δ swi4Δ GAL- BCK2**
cln3Δ swi4Δ whi5Δ*
mbp1Δ swi4Δ GAL- BCK2
*mbp1Δ swi4Δ GAL- CLB5**
mbp1Δ swi4Δ GAL-CLN2
*mbp1Δ swi4Δ GAL-CLN3**
*mbp1Δ swi4Δ cdh1Δ**
*mbp1Δ swi4Δ sic1Δ**
*mbp1Δ swi4Δ whi5Δ**
swi4Δ swi6Δ sic1Δ
swi4Δ swi6Δ GAL- BCK2
*swi4Δ swi6Δ GAL-CLB5**
swi4Δ swi6Δ GAL-CLN2
swi4Δ swi6Δ GAL-CLN3
*swi4Δ swi6Δ whi5Δ**
*bck2Δ cln3Δ mbp1Δ whi5Δ**
bck2Δ cln3Δ swi4Δ whi5Δ
bck2Δ cln3Δ swi6Δ GAL-CLN3
cln1Δ cln2Δ cln3Δ whi5Δ
cln3Δ swi4Δ whi5Δ sic1Δ*

Bck2 mutants
bck2Δ
Multi-copy *BCK2*
*bck2Δ cln3Δ cdh1Δ**
bck2Δ cln3Δ sic1Δ
*bck2Δ cln3Δ GAL-CLB5**
bck2Δ cln3Δ multi-copy *CLN2*
bck2Δ cln3Δ GAL-CLN3
*bck2Δ cln3Δ cdc6Δ sic1Δ**
bck2Δ cln3Δ GAL-CLN2

Cln mutants
cln3Δ
GAL-CLN2
GAL-CLN3

cln1Δ cln2Δ
cln1Δcln2Δ bck2Δ
cln1Δ cln2Δ cdh1Δ
cln1Δ cln2Δ cln3Δ
cln1Δ cln2Δ sic1Δ
cln1Δ cln2Δ GAL-SIC1
cln1Δ cln2Δ cdc6Δ *sic1Δ*
cln1Δ cln2Δ bck2Δ cdh1Δ*
cln1Δ cln2Δ cdh1Δ GAL-CLN2
cln1Δ cln2Δ cdh1Δ GAL-SIC1
cln1Δ cln2Δ cln3Δ apc^{ts}
cln1Δ cln2Δ cln3Δ cdh1Δ
cln1Δ cln2Δ cln3Δ sic1Δ
cln1Δ cln2Δ cln3Δ multi-copy *BCK2*
cln1Δ cln2Δ cln3Δ GAL-CLB2
cln1Δ cln2Δ cln3Δ GAL-CLB5
cln1Δ cln2Δ cln3Δ multi-copy *CLB5*
cln1Δ cln2Δ cln3Δ GAL-CLN2
cln1Δ cln2Δ cln3Δ GAL-CLN3
cln1Δ cln2Δ GAL-SIC1 GAL-CLN2
*cln1Δ cln2Δ cln3Δ sic1Δ cdc6Δ**
cln1Δ cln2Δ GAL-SIC1 GAL-CLN2 cdh1Δ
cln1Δ cln2Δ cln3Δ bck2Δ GAL-CLN2

Cdh1, Sic1 mutants
cdh1Δ
cdc6Δ
sic1Δ
swi5Δ
GAL-CDC6
Multi-copy *CDC6*
CDH1 constitutively active
SIC1-4A
GAL-SIC1
Multi-copy *SIC1*
Multi-copy *SIC1-high*
GAL-SIC1-dbΔ
GAL-SIC1-4A
cdc6Δ sic1Δ
sic1Δ GAL-CLB2
cdh1Δ GAL-CLB2
swi5Δ GAL-CLB2
cdh1Δ GAL-CLB5
sic1Δ GAL-CLB5
sic1Δ CLB5-dbΔ
cdc6Δ sic1Δ cdh1Δ
sic1Δ cdh1Δ GAL-CDC20
swi5Δ cdh1Δ GAL-SIC1
SIC1-4A clb5Δ clb6Δ
sic1Δ cdc6Δ cdh1Δ GAL-CDC20

Clb5 Clb6 mutants
clb5Δ
GAL-CLB5
Multi-copy *CLB5*
CLB5-dbΔ
GAL-CLB5-dbΔ
clb5Δ clb6Δ
clb5Δ pds1Δ
CLB5-dbΔ pds1Δ
clb5Δ clb6Δ pds1Δ
clb5Δ pds1Δ* + multi-copy *SIC1-high
clb5Δ clb6Δ cln1Δ cln2Δ
clb5Δ clb6Δ pds1Δ* + multi-copy *SIC1-high

Clb1 Clb2 mutants
GAL-CLB2
CLB2-dbΔ
***CLB2-dbΔ* in galactose**
Multi-copy *GAL-CLB2*
clb2Δ CLB1
CLB2-dbΔ clb5Δ
***CLB2-dbΔ clb5Δ* in gal**
CLB2-dbΔ GAL-SIC1
CLB2-dbΔ multi-copy *SIC1*
clb2Δ CLB1 cdh1Δ

clb1Δ clb2Δ clb5Δ
clb2Δ CLB1 pds1Δ
CLB2-dbΔ clb5Δ clb6Δ
CLB2-dbΔ clb5Δ clb6Δ* in gal
clb1Δ clb2Δ clb5Δ clb6Δ

Cdc20 mutants
cdc20^{ts}
GAL-CDC20
cdc20Δ clb5Δ
cdc20Δ pds1Δ
cdc20Δ GAL-SIC1-4A
cdc20Δ clb5Δ pds1Δ
cdc20Δ pds1Δ cdh1Δ
cdc20Δ pds1Δ CLB5-dbΔ
*cdc20Δ pds1Δ SIC1-4A**
cdc20Δ pds1Δ+ multi-copy *SIC1-high*
cdc20Δ clb5Δ clb6Δ pds1Δ
cdc20Δ pds1Δ clb5Δcdh1Δ
cdc20Δ pds1Δ cdh1Δ+ multi-copy *SIC1-high*
cdc20Δ pds1Δ clb5Δ + multi-copy *SIC1-high*
cdc20Δ pds1Δ clb5Δ cdh1Δ + multi-copy *SIC1-high*

APC mutants
APC-A
APC-A cdh1Δ
APC-A cdh1Δ in galactose
APC-A sic1Δ
APC-A GAL-CLB2
APC-A cdc6Δ sic1Δ
APC-A cdh1Δ GAL-CDC6
APC-A cdh1Δ multi-copy *CDC6*
APC-A cdh1Δ GAL-SIC1
APC-A cdh1Δ multi-copy *SIC1*
APC-A chd1Δ multi-copy *CDC20*

Pds1/Esp1 interaction
cdc55Δ
cdc55Δ in galactose
esp1^{ts}
pds1Δ
pds1Δ in galactose
GAL-CDC55
PDS1-dbΔ
GAL-PDS1-dbΔ
GAL-ESP1 cdc20^{ts}
GAL-PDS1-dbΔ esp1^{ts}
GAL-ESP1 GAL-PDS1-dbΔ
cdc20Δ GAL-PDS1-dbΔ GAL-TEV CDC20-back
GAL-TEV cdc20Δ
GAL-ESP1cdc20Δ clb5Δ
GAL-PDS1-dbΔ GAL-TEV cdc20-back
GAL-TEV cdc20Δ clb5Δ
GAL-TEV cdc20Δ GAL- SIC1

Checkpoint mutants
WT in hydroxyurea
WT in nocodazole
bub2Δ
bub2Δ in nocodazole
bub2Δ in hydroxyurea-arrested cells
mad2Δ
mad2Δ in nocodazole
net1^{ts} in nocodazole
pds1Δ in nocodazole
bub2Δ cdc20^{ts}
bub2Δ pds1Δ in nocodazole
mad2Δ bub2Δ
mad2Δ bub2Δ in nocodazole
mad2Δ cdc20^{ts}
mad2Δ pds1Δ in nocodazole
mad2Δ GAL-TEM1 in nocodazole

MEN pathway mutants
cdc15Δ
cdc15^{as1}
lte1Δ
tem1Δ
GAL-CDC15 in hydroxyurea-arrested cells *GAL-CDC15*
Multi-copy *CDC15*

GAL-TEM1
multi-copy *TEM1*
cdc15Δ net1^{ts}
cdc15Δ cdc20-back
lte1Δ bub2Δ
lte1Δ esp1^{ts}
tem1Δ net1^{ts}
cdc15^{ts} multi-copy *CDC14*
cdc15Δ GAL-SIC1
cdc15^{ts} multi-copy *TEM1*
cdc15Δ* multi-copy *SWI5
tem1^{ts} *GAL-CDC15*
tem1Δ multi-copy *CDC14*
tem1^{ts} multi-copy *CDC15*
cdc15Δ net1^{ts} *cdh1Δ*
cdc15^{as1} *cdc20Δ pds1Δ*
cdc15^{as1} *cdc20Δ pds1Δ clb5Δ*
cdc15^{as1} *cdc20Δ pds1Δ clb5Δ clb6Δ*

Exit-from-mitosis mutants
cdc5Δ
cdc14^{ts}
cdc14^{ts} *in galactose*
net1^{ts}
swe1Δ
CDC5-dbΔ
GAL-CDC5
Nocodazole then *GAL-CDC5*
Hydroxyurea then *GAL-CDC5*
GAL-CDC14
GAL-NET1
TAB6-1
cdc5Δ bub2Δ
cdc5Δ cdc15Δ
cdc5Δ net1Δ
cdc5Δ tem1Δ
cdc14^{ts} *cdh1Δ* non-permissive temperature
cdc14^{ts} *sic1Δ* non- permissive temperature
net1^{ts} *cdc20*^{ts}
cdc5Δ GAL-CDC15
cdc5Δ GAL-SIC1
cdc5Δ multi-copy *SWI5*
cdc14-1 GAL-CDC15
bub2Δ GAL-CDC5 in hydroxyurea
GAL-CDC5 cdc15Δ
cdc14^{ts} *GAL-CLN2* non-permissive temp.
cdc14^{ts} *GAL-SIC1*
TAB6-1 cdc15^{ts}
TAB6-1 clb5Δ
GAL-CDC5 cdc20Δ
GAL-CDC14 GAL-NET1
TAB6-1 clb5Δ clb6Δ
TAB6-1 clb2Δ CLB1
*GAL-CDC5 GAL-PDS1 cdc20Δ**
cdc20Δ then *GAL-PDS1dbΔ GAL-CDC5*
cdc20Δpds1Δ cdh1Δ then *URL-cdc5*

***Cdc14- oscillating* mutants**
GAL-CLB2-dbΔ
GAL-CLB2nd
GAL-CLB2-dbΔ cdc5-1
GAL-CLB2-dbΔ CDC5-dbΔ
GAL-CLB2-dbΔ cdc14-1
GAL-CLB2-dbΔ cdc15^{as1}
GAL-CLB2-dbΔ cdc20-3
GAL-CLB2-dbΔ cdh1Δ
GAL-CLB2-dbΔ sic1Δ
GAL-CLB2-dbΔ swi5Δ
GAL-CLB2-dbΔ cdc6Δ *sic1Δ**
*GAL-CLB2-dbΔ cdc20Δ clb5Δ pds1Δ**
GAL-CLB2nd *cdc5*-1
GAL-CLB2nd *CDC5-dbΔ*
GAL-CLB2nd *cdc14*-1
GAL-CLB2nd *cdc15*^{as1}
GAL-CLB2nd *cdc20-3*
GAL-CLB2nd *cdh1Δ*
GAL-CLB2nd *sic1Δ*
GAL-CLB2nd *swi5Δ*
GAL-CLB2ndcdc6Δ *sic1Δ**
GAL-CLB2ndcdc20Δ *clb5Δ pds1Δ**

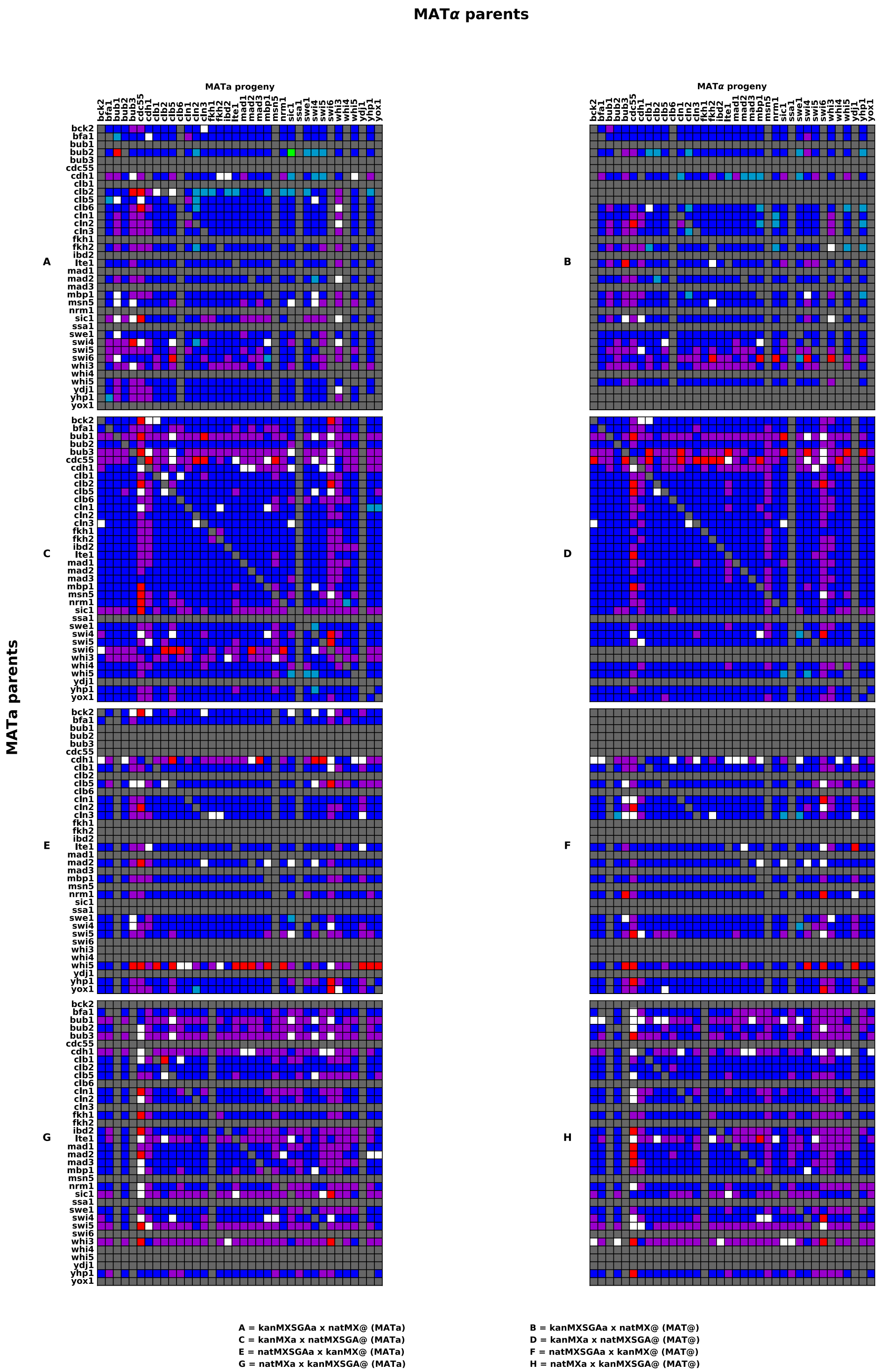

Figure S6. Comparison of fitness scores for double mutants in all four sets of crosses on YPR media. White cells indicate zero growth and grey cells indicate missing or excluded data. Royal blue is used to designate fitness scores that differ from WT by fewer than 2 standard deviations. Cyan and green indicate fitness scores that are greater than WT by up to or more than 6 standard deviations respectively. Magenta and red indicate fitness scores that are less than WT by up to or more than 6 standard deviations respectively. **A & B) Cross 1. C & D) Cross 2. E & F) Cross 3. G & H) Cross 4.**

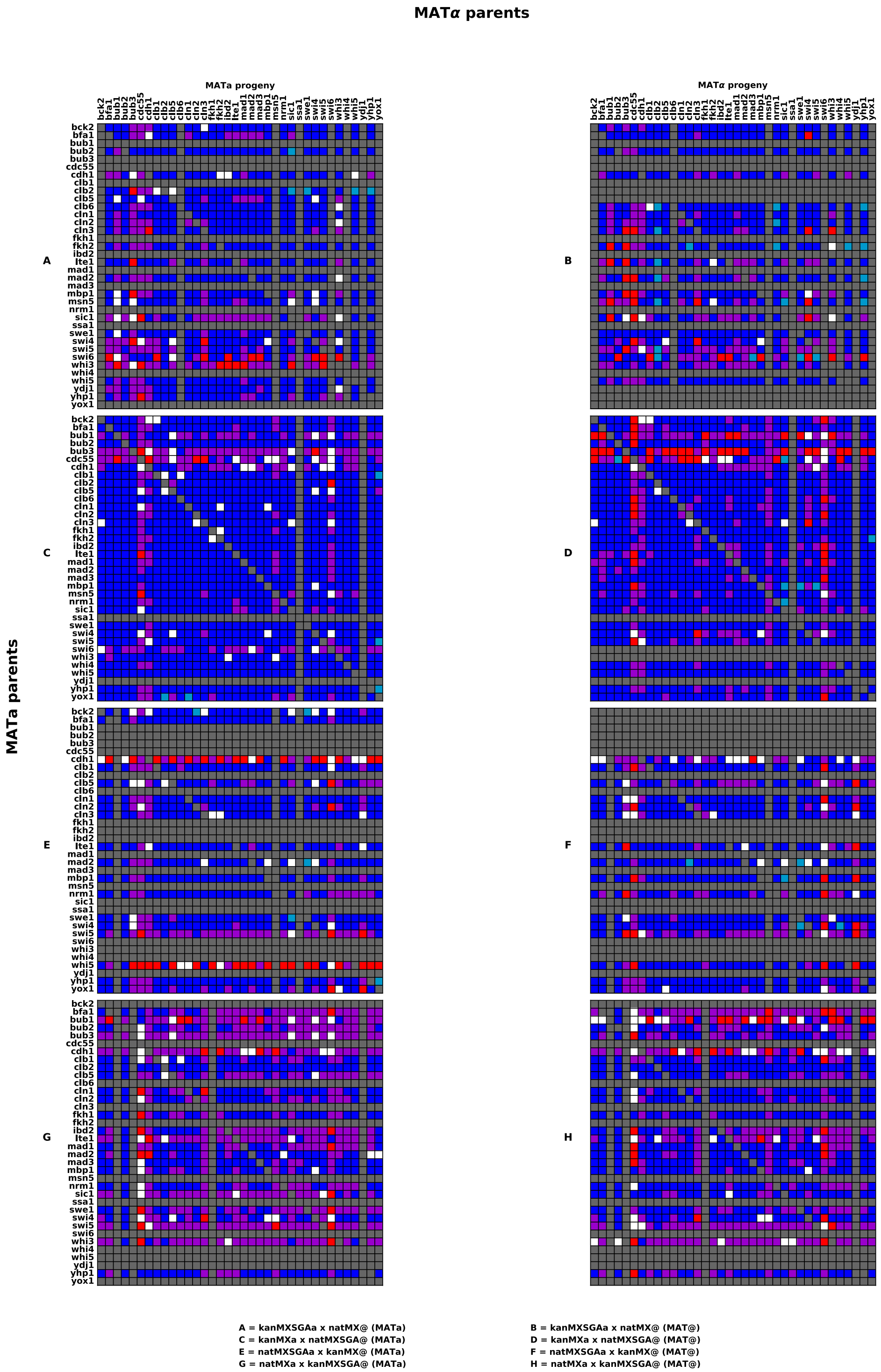

Figure S7. Comparison of fitness scores for double mutants in all four sets of crosses on YPG media. White cells indicate zero growth and grey cells indicate missing or excluded data. Royal blue is used to designate fitness scores that differ from WT by fewer than 2 standard deviations. Cyan and green indicate fitness scores that are greater than WT by up to or more than 6 standard deviations respectively. Magenta and red indicate fitness scores that are less than WT by up to or more than 6 standard deviations respectively. **A & B) Cross 1. C & D) Cross 2. E & F) Cross 3. G & H) Cross 4.**

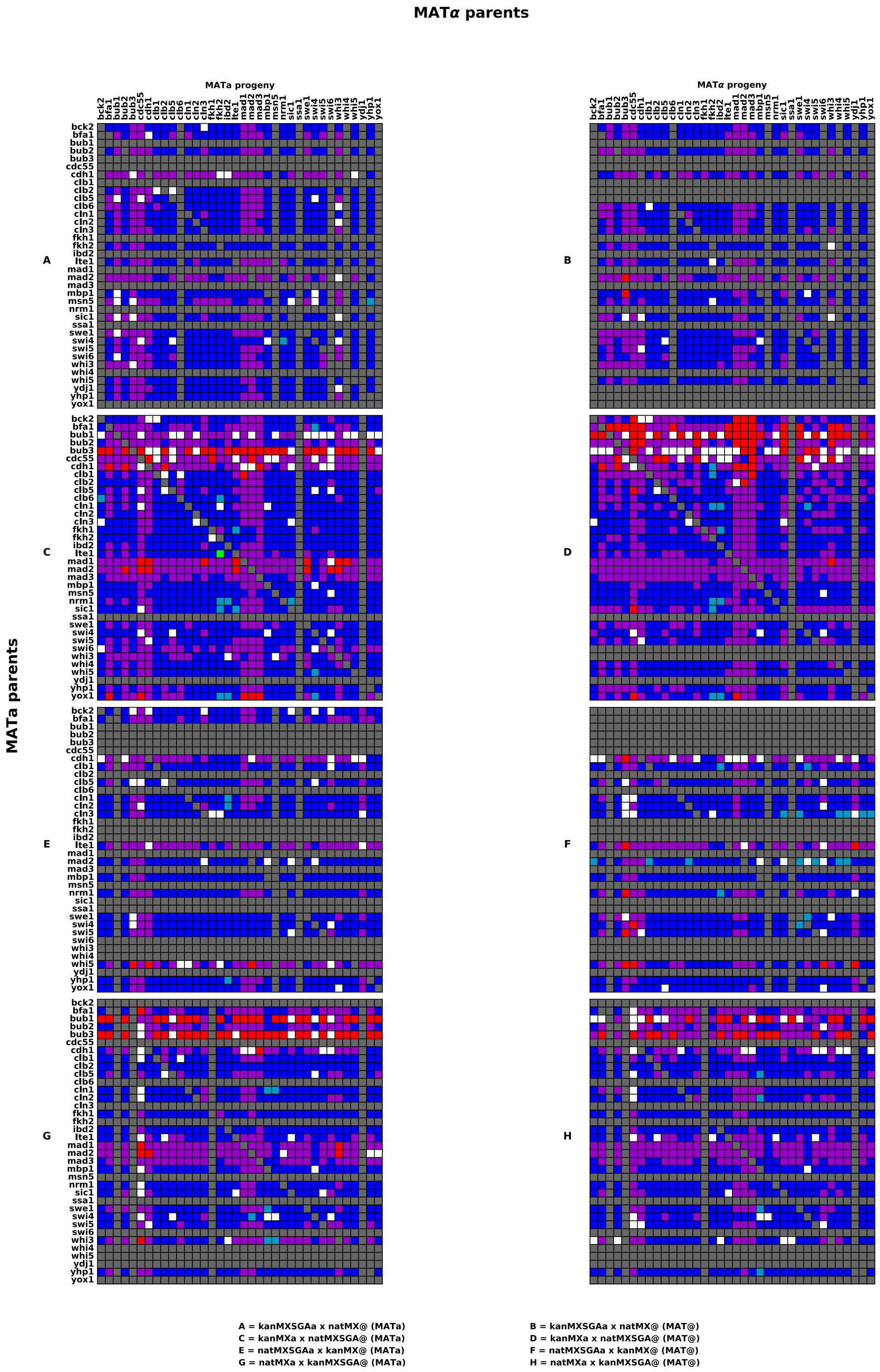

Figure S8. Comparison of fitness scores for double mutants in all four sets of crosses on YPD-Ben media. White cells indicate zero growth and grey cells indicate missing or excluded data. Royal blue is used to designate fitness scores that differ from WT by fewer than 2 standard deviations. Cyan and green indicate fitness scores that are greater than WT by up to or more than 6 standard deviations respectively. Magenta and red indicate fitness scores that are less than WT by up to or more than 6 standard deviations respectively. **A & B) Cross 1. C & D) Cross 2. E & F) Cross 3. G & H) Cross 4.**

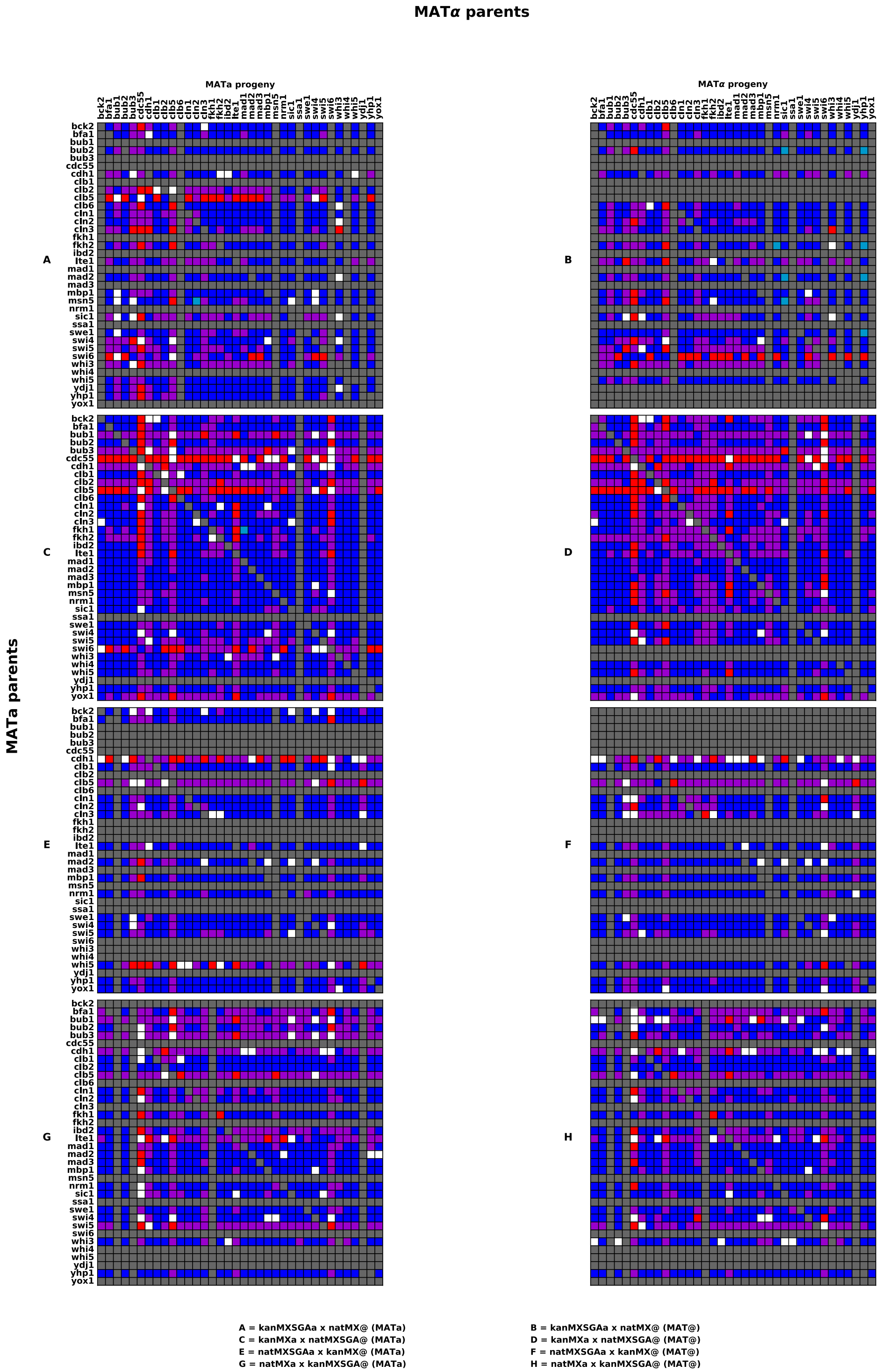

Figure S9. Comparison of fitness scores for double mutants in all four sets of crosses on YPD-CPT media. White cells indicate zero growth and grey cells indicate missing or excluded data. Royal blue is used to designate fitness scores that differ from WT by fewer than 2 standard deviations. Cyan and green indicate fitness scores that are greater than WT by up to or more than 6 standard deviations respectively. Magenta and red indicate fitness scores that are less than WT by up to or more than 6 standard deviations respectively. **A & B) Cross 1. C & D) Cross 2. E & F) Cross 3. G & H) Cross 4.**

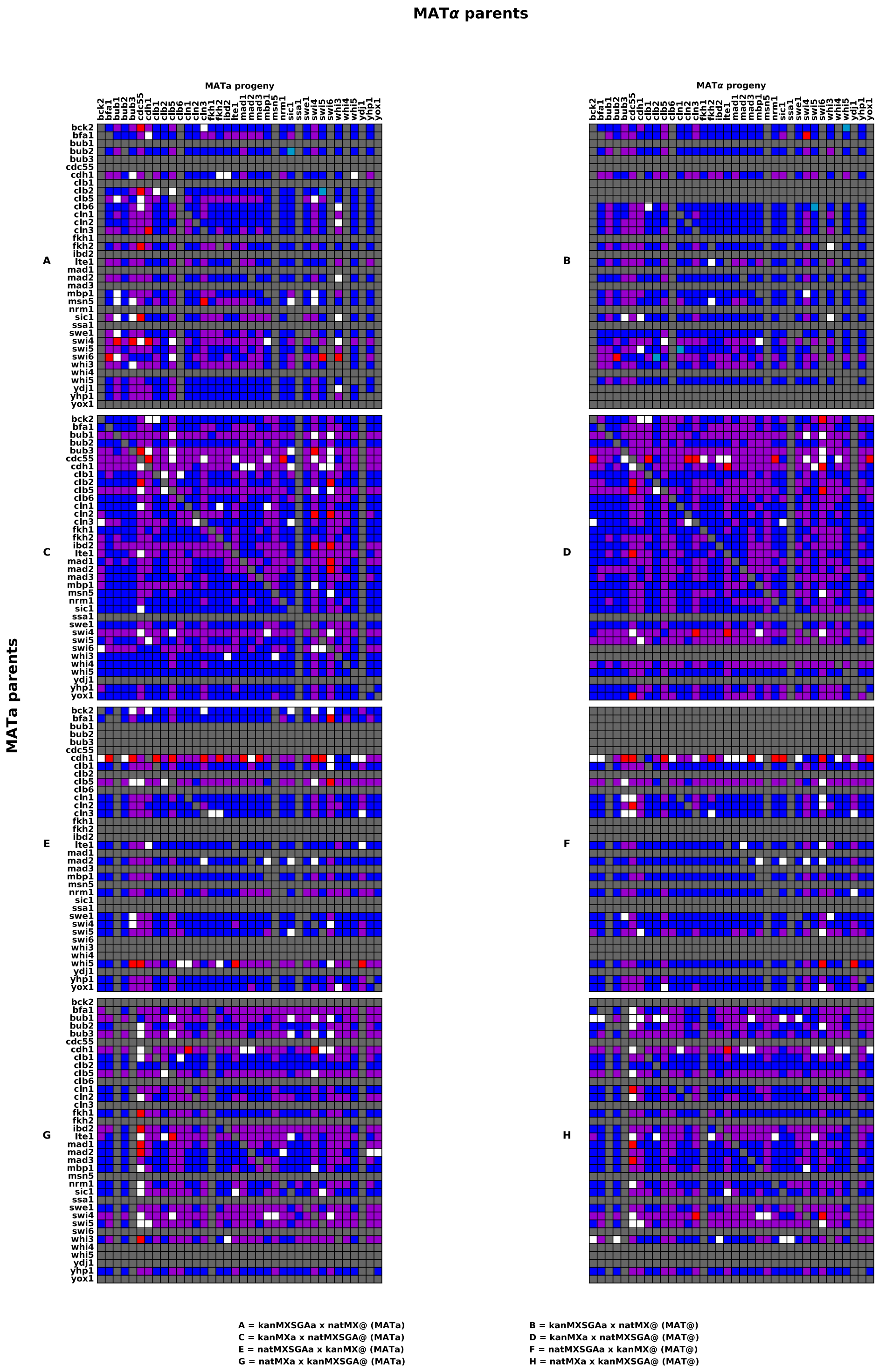

Figure S10. Comparison of fitness scores for double mutants in all four sets of crosses on YPD-HU media. White cells indicate zero growth and grey cells indicate missing or excluded data. Royal blue is used to designate fitness scores that differ from WT by fewer than 2 standard deviations. Cyan and green indicate fitness scores that are greater than WT by up to or more than 6 standard deviations respectively. Magenta and red indicate fitness scores that are less than WT by up to or more than 6 standard deviations respectively. **A & B) Cross 1. C & D) Cross 2. E & F) Cross 3. G & H) Cross 4.**

Supplementary Tables

Supplementary tables are provided as Excel files in the Supplementary Data file.

Table S1. Parent Strains used in this study

Table S2. Primers used in this study

Table S3. Potential sources of false negatives

Table S4. Replicates supporting synthetic lethal (SL)

Table S5. Colony size and growth rate variability across biological replicates of *cdh1Δ swi4Δ*

Supplementary References

1 Giaever, G. *et al.* Functional profiling of the Saccharomyces cerevisiae genome. *Nature* **418**, 387-391 (2002).

2 Sherman, F. & Hicks, J. in *Guide to yeast genetics and molecular biology* Vol. 194 *Methods Enzymol* (ed Christine Guthrie and Gerald R. Fink) Ch. 3 21-37 (Academic Press, 1991).

3 Goldstein, A. L. & McCusker, J. H. Three new dominant drug resistance cassettes for gene disruption in Saccharomyces cerevisiae. *Yeast* **15**, 1541-1553 (1999).

4 Knop, M. *et al.* Epitope tagging of yeast genes using a PCR-based strategy: more tags and improved practical routines. *Yeast* **15**, 963-972 (1999).

5 Longtine, M. S. *et al.* Additional modules for versatile and economical PCR-based gene deletion and modification in Saccharomyces cerevisiae. *Yeast* **14**, 953-961 (1998).

6 Schiestl, R. H. & Gietz, R. D. High efficiency transformation of intact yeast cells using single stranded nucleic acids as a carrier. *Curr Genet* **16**, 339-346 (1989).

7 Tong, A. H. & Boone, C. Synthetic genetic array analysis in Saccharomyces cerevisiae. *Methods Mol Biol* **313**, 171-192 (2006).

8 OpenWetWare. *Endy:Yeast Colony PCR*, <https://openwetware.org/mediawiki/index.php?title=Endy:Yeast_Colony_PCR&oldid=348785> (2009).

9 Sprague, G. F., Jr. Assay of yeast mating reaction. *Methods Enzymol* **194**, 77-93 (1991).

10 Hsu, W. S. *et al.* S-phase cyclin-dependent kinases promote sister chromatid cohesion in budding yeast. *Molecular and cellular biology* **31**, 2470-2483 (2011).

11 Stuart, D. & Wittenberg, C. CLB5 and CLB6 are required for premeiotic DNA replication and activation of the meiotic S/M checkpoint. *Genes Dev* **12**, 2698-2710 (1998).

12 Segal, M., Clarke, D. J. & Reed, S. I. Clb5-associated kinase activity is required early in the spindle pathway for correct preanaphase nuclear positioning in Saccharomyces cerevisiae. *The Journal of cell biology* **143**, 135-145 (1998).

13 McCourt, P., Gallo-Ebert, C., Gonghong, Y., Jiang, Y. & Nickels, J. T., Jr. PP2A(Cdc55) regulates G1 cyclin stability. *Cell Cycle* **12**, 1201-1210 (2013).

14 Loeb, J. D., Kerentseva, T. A., Pan, T., Sepulveda-Becerra, M. & Liu, H. Saccharomyces cerevisiae G1 cyclins are differentially involved in invasive and pseudohyphal growth independent of the filamentation mitogen-activated protein kinase pathway. *Genetics* **153**, 1535-1546 (1999).

15 Oehlen, L. J. & Cross, F. R. Potential regulation of Ste20 function by the Cln1-Cdc28 and Cln2-Cdc28 cyclin-dependent protein kinases. *J Biol Chem* **273**, 25089-25097 (1998).

16 Levine, K., Huang, K. & Cross, F. R. Saccharomyces cerevisiae G1 cyclins differ in their intrinsic functional specificities. *Molecular and cellular biology* **16**, 6794-6803 (1996).

17 Cross, F. R. Cell cycle arrest caused by CLN gene deficiency in Saccharomyces cerevisiae resembles START-I arrest and is independent of the mating-pheromone signalling pathway. *Molecular and cellular biology* **10**, 6482-6490 (1990).

18 Cvrcková, F. & Nasmyth, K. Yeast G1 cyclins CLN1 and CLN2 and a GAP-like protein have a role in bud formation. *The EMBO journal* **12**, 5277-5286 (1993).

19 Mitchell, D. A. & Sprague, G. F., Jr. The phosphotyrosyl phosphatase activator, Ncs1p (Rrd1p), functions with Cla4p to regulate the G(2)/M transition in Saccharomyces cerevisiae. *Molecular and cellular biology* **21**, 488-500 (2001).

20 McCourt, P., Gallo-Ebert, C., Gonghong, Y., Jiang, Y. & Nickels, J. T., Jr. PP2A(Cdc55) regulates G1 cyclin stability. *Cell cycle (Georgetown, Tex.)* **12**, 1201-1210 (2013).

21 Alepuz, P. M., Matheos, D., Cunningham, K. W. & Estruch, F. The Saccharomyces cerevisiae RanGTP-binding protein msn5p is involved in different signal transduction pathways. *Genetics* **153**, 1219-1231 (1999).

22 Pic, A. *et al.* The forkhead protein Fkh2 is a component of the yeast cell cycle transcription factor SFF. *EMBO J* **19**, 3750-3761 (2000).

23 Becker, J., Walter, W., Yan, W. & Craig, E. A. Functional interaction of cytosolic hsp70 and a DnaJ-related protein, Ydj1p, in protein translocation in vivo. *Molecular and cellular biology* **16**, 4378-4386 (1996).
